# Performance analysis and structural optimization of drill pipe connections under complex operating conditions

**DOI:** 10.1371/journal.pone.0355727

**Published:** 2026-08-13

**Authors:** Wanlong Huang, Yinghong Zou, Chunjie Song, Xiang Zeng

**Affiliations:** 1 College of Agriculture and Forestry Engineering and Planning, Tongren University, Tong Ren, Guizhou, China; 2 College of Art, Tongren University, Tong Ren, Guizhou, China; Industrial University of Ho Chi Minh City, VIET NAM

## Abstract

In order to reduce the failure of thread joint of drill pipe, its connection performance and sealing performance need to be improved. Based on the mechanical analysis of the joint and yield criterion of von Mises, the three-dimensional finite element model of the single shoulder and double shoulder with NC38 thread profile was established. The failure mechanism under the combined working conditions is discussed by combining test and numerical simulation. A new type joint of TM40 is further proposed, and the mechanical properties of the shoulder and thread section of the joint are compared and analyzed. The results show that the stress of the double shoulder structure is significantly lower than that of the single shoulder structure, and the sealing performance is better. At the same time, the extension threaded joint of TM40 can improve the stress distribution of the threaded section, reduce the stress concentration, ensure the reasonable distribution of the contact pressure on the shoulder.

## 1. Introduction

The strength and stability of the drill pipe connection is a crucial factor in drilling engineering. During the drilling process, the connection part bears a complex mechanical environment, including torque, tensile load and bending load, which will lead to the fatigue and failure of the connection [1–3]. Therefore, in-depth stress analysis of threaded connections is essential to ensure their reliability and safety under complex working conditions [4,5]. Researchers have carried out extensive research in this field to improve the drilling efficiency. Liu et al. [6] established a three-dimensional model with thread rise angle to study the self-loosening behavior of threaded connections. The study showed that the relative slip and plastic deformation of internal and external threads caused by the torsional deformation of threads were the main reasons for the self-loosening of threaded connections. Deng et al. [7,8] used the power exponent hardening constitutive model to theoretically analyze the tightening process of bolt connection, and established a refined finite element model of threaded connection for verification. The analysis showed that the conversion efficiency of tightening axial force could be improved by reducing the friction coefficient of threaded connection pair. Shi et al. [9] established a comprehensive model of threaded connection considering tension torsion coupling, and modified the thread deformation theory to analyze the influence of the load distribution of threaded connection on its static and dynamic performance. G. Dinger [10] evaluated the assembly process of thread forming by combining numerical simulation with experiment, which could better understand its forming mechanism and reduce experimental testing. Di et al. [11,12] studied the mechanical properties of drill tool joint under the action of combined load through three-dimensional finite element model, and found that the bending moment would lead to the asymmetric distribution of joint stress. Shi et al. [13] established the force analysis model of different threads in the expansion process, and studied the influence of thread deformation on sealing performance during expansion by optimizing thread parameters. Amir et al. [14] studied and discussed the influence of the geometry of the screw tooth on the stress propagation by combining the experimental and numerical simulation methods, and found that increasing the tooth height and number of the screw tooth can reduce the fluctuation level of the stress. Jia et al. [15,16] studied the effects of pitch, pre tightening force and thread connection length on the axial stress distribution of threads, and found that the stress distribution was more uniform, and the fatigue strength and service life were significantly improved after Spiralock thread locking. Dong et al. [17] optimized the key thread parameters based on the safety evaluation method of fatigue life to reduce the vibration fatigue of the union to reduce the risk of fracturing manifold failure. Zhang et al. [18,19] established a three-dimensional model with thread lift angle, studied the load transfer behavior of meshing thread under axial tensile load, and reasonably reflected the actual situation based on the formula modified by Yamamoto theory.

The above documents mainly focus on the research of large-size drill pipe with a diameter of more than 5.5inch, and less on the design and research of drill pipe joints with a diameter of less than 4.5inch. The drill pipe with small size under the combined load is prone to failure in the slim hole location of ultra short radius. It is necessary to study the effect of external load on high-strength drill pipe joints. This paper optimizes the conventional thread joints based on drilling safety considerations, material tests and simulation calculations, and changes its structure to obtain higher strength drill pipe joints.

## 2. Test and analysis of material performance

### 2.1. Tensile property test

In the practical application of threaded joints, the tensile properties of materials are directly related to the bearing capacity and service life of threaded joints, so the material properties of the joint can be better evaluated through tensile tests [20].

**Fig 1:**
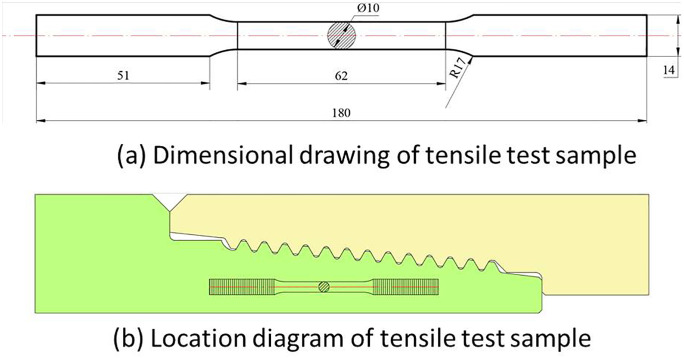
Schematic diagram of joint tensile specimen sampling. (a) Dimensional drawing of tensile test sample, (b) Location diagram of tensile test sample.

**Fig 2:**
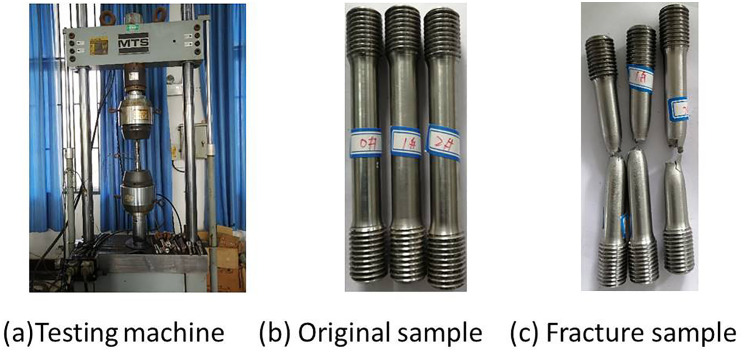
Results of tensile test. (a)Testing machine,(b) Original sample,(c) Fracture sample.

In the test, refer to metallic materials tensile test method at room temperature of GB/T 228–2002. Three round rod tensile samples (with a gauge section diameter of Φ 10 mm) were prepared from the threaded joint material of drill pipe. The sampling diagram is shown in Fig 1. MTS testing machine was used to test the mechanical properties of the tensile samples taken from the joint part in Fig 2. The test results are shown in Table 1.

**Table 1:** Tensile mechanical performance test results.

Number	Yield strength/MPa		Tensile strength/MPa		Elongation/%	
	Measured value	Mean value	Measured value	Mean value	Measured value	Mean value
Sample1	861	862	973	978	22.24	21.98
Sample2	858		978		21.79	
Sample3	867		983		21.82	

**Fig 3:**
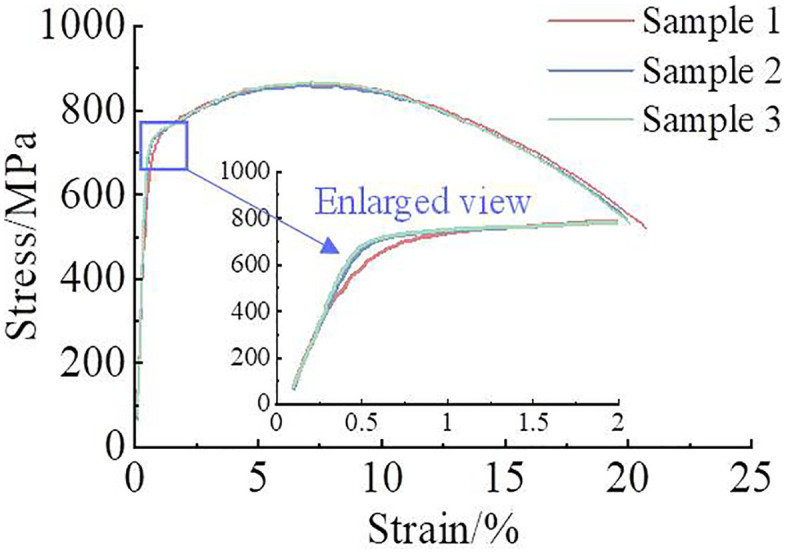
Stress strain curves of tensile test.

According to the stress-strain curves in Fig 3, the stress-strain curves of the three samples at the elastic stage are basically the same. There is an obvious inflection point when the curves reach the yield point. The three samples show obvious yield phenomenon, and then the stress gradually increases, and the material enters the plastic deformation stage, until reaching the maximum tensile strength, with further stretching, the material breaks. The enlarged drawing shows the stress-strain relationship between the elastic stage and the yield stage, which can more clearly observe the stress-strain changesof the material at the yield point.

### 2.2. Impact performance test

**Fig 4:**
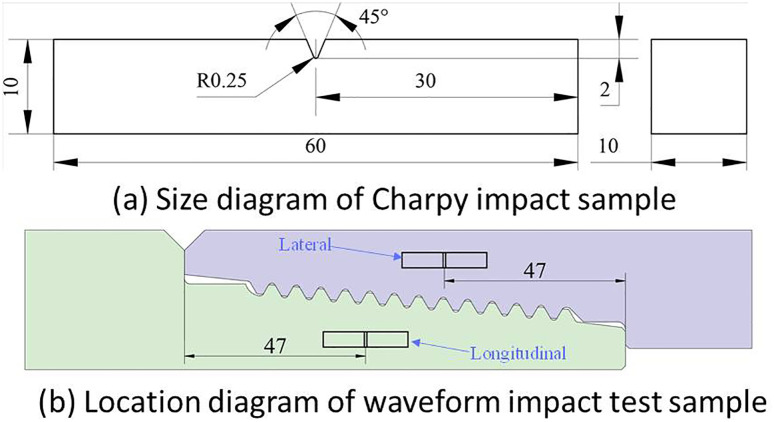
Schematic diagram of impact sample of joint. (a) Size diagram of Charpy impact sample, (b) Location diagram of joint waveform impact test sample.

**Fig 5:**
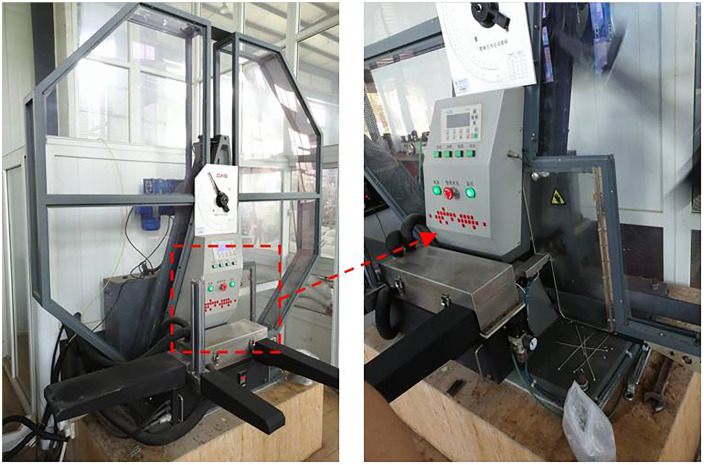
High and low temperature wave shock tester of fully automatic.

According to the standard of Charpy pendulum impact test method for metallic materials of GB/T 229−2007, the impact performance test of the material at the threaded joint of drill pipe was carried out under the conditions of normal temperature (25°C), low temperature (−20°C) and ultra-low temperature (−60°C). The influence law of low temperature environment on the impact properties of threaded joints is revealed by comparing and analyzing the impact properties of materials under different temperature conditions, which provides data support and theoretical basis for the safety design and failure prevention of drilling tools under low temperature environment. The impact sample size is shown in Fig 4(a), and the longitudinal and transverse impact samples of the joint are taken from the position of the threaded joint as shown in Fig 4(b), Fig 5 is the photos of high and low temperature wave shock tester of fully automatic.

**Fig 6:**
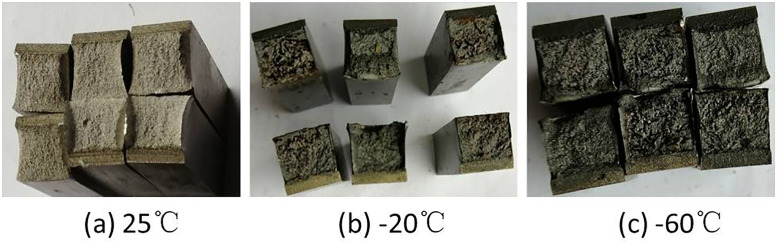
Photos of the impact fracture surface of the impact sample. (a) 25°C,(b) −20°C,(c) −60°C.

As shown in Fig 6, the impact fracture surface is relatively smooth at room temperature (25°C), and the fracture form is relatively uniform, indicating that the material has high toughness and strong resistance to impact fracture at room temperature. At low temperature (−20°C), the impact fracture surface appears irregular shape, with obvious oblique section, and the fracture form is relatively brittle, indicating that the toughness of the material decreases under low temperature conditions, and brittle fracture is easy to occur. At ultra-low temperature (−60°C), the fracture surface is very rough and the crack propagation inside the material is obvious. This fracture morphology shows that the material shows strong brittleness at extremely low temperature, and the toughness is almost lost, and it is more prone to brittle fracture when bearing impact load. The toughness of the material gradually decreases with the decrease of temperature, and the fracture form changes from ductile fracture to brittle fracture. At the same time, the surface energy of the fracture surface is higher due to rapid fracture, which reduces the surface gloss, and the surface color of the material is darker.

**Fig 7:**
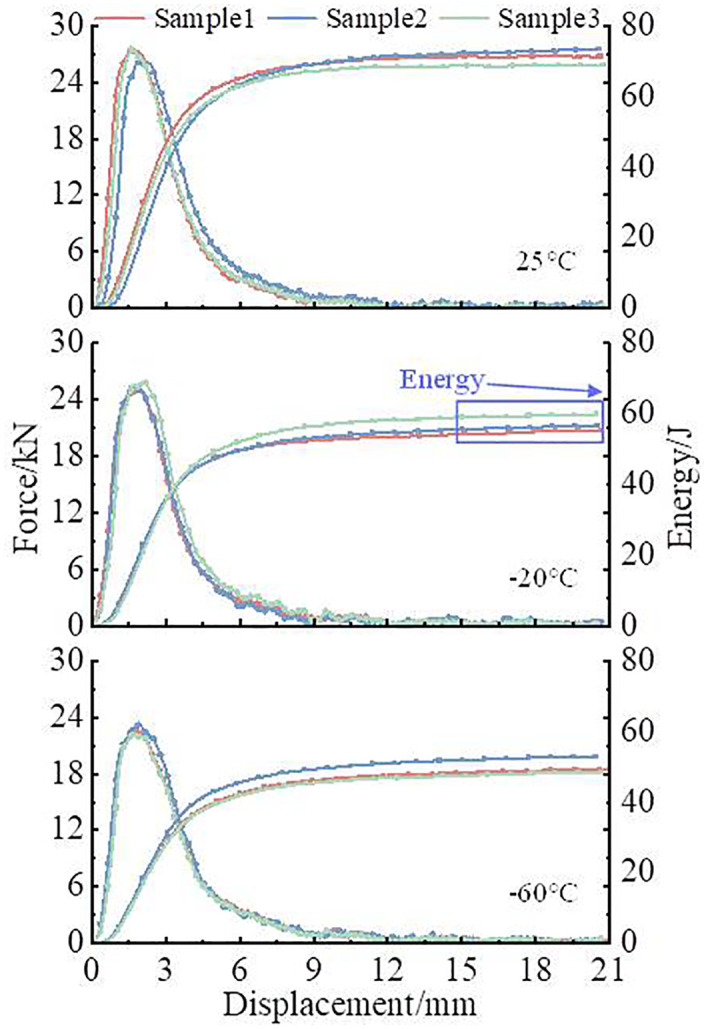
Transverse shock curves.

**Fig 8:**
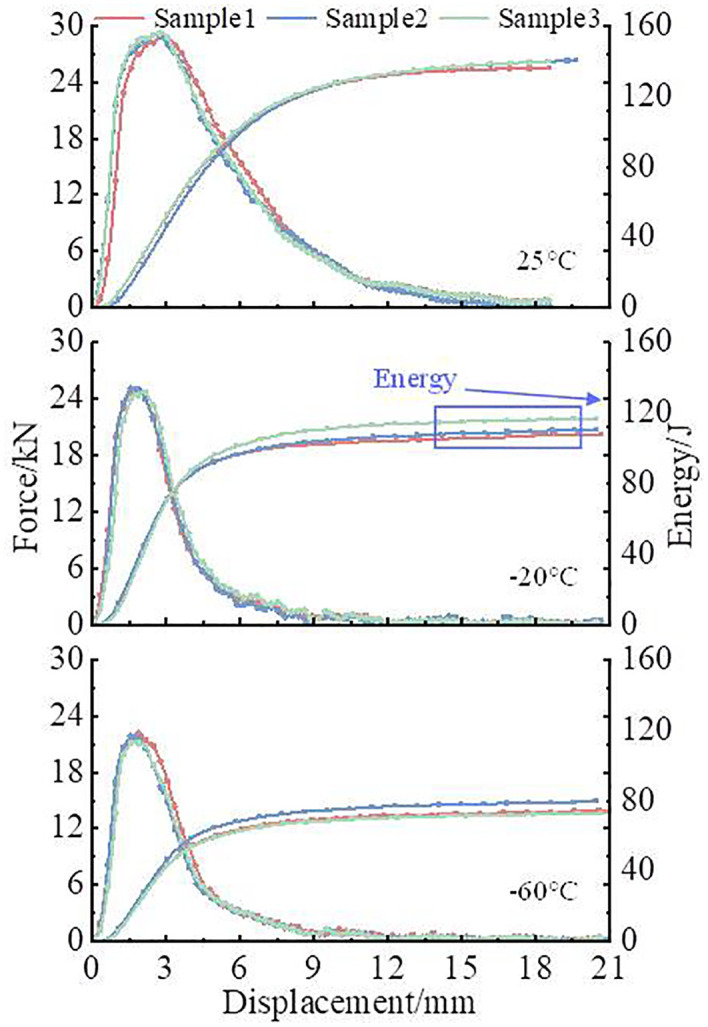
Longitudinal shock curves.

Table 2 and Table 3 show the parameters of the oscillographic impact test results of the transverse and longitudinal specimens at normal temperature (25 °C), low temperature (−20 °C) and ultra-low temperature (−60 °C), and Fig 7 and Fig 8 show the oscillographic impact curves.

**Table 2:** Results of transverse wave shock experiment with various parameters.

*T*	S/N	*W*_*t*_ (J)	*S*_*t*_(mm)	*F*_*m*_(kN)	*S*_*m*_ (mm)	*F*_*gy*_(kN)	*S*_*gy*_(mm)	*W*_*m*_(J)	*W*_*a*_(J)
25°C	1	79.82	10.72	17.24	0.846	15.84	0.669	7.02	72.8
	2	79.55	11.9	21.31	1.883	18.19	1.076	23.81	55.74
	3	73.33	11.99	20.47	1.683	18.31	1.18	17.52	55.81
	Average	77.57	11.54	19.67	1.47	17.45	0.98	16.12	61.45
−20°C	1	74.1	11.74	21.88	1.567	19.28	1.024	19.09	55.01
	2	75.91	11.96	20.95	1.882	18.19	1.369	17.64	58.27
	3	71.28	11.14	22.1	1.632	19.21	1.159	17.84	53.44
	Average	73.76	11.61	21.64	1.69	18.89	1.18	18.19	55.57
−60°C	1	71.53	8.87	22.23	1.66	19.16	1.086	19.96	51.57
	2	74.88	12.22	22.12	1.949	18.62	1.013	27.1	47.78
	3	77.21	9.04	22.82	2.106	18.91	1.18	27.81	49.4
	Average	74.54	10.04	22.39	1.91	18.90	1.09	24.96	49.58

**Table 3:** Parameters of longitudinal wave shock experiment results.

*T*	S/N	*W*_*t*_ (J)	*S*_*t*_(mm)	*F*_*m*_(kN)	*S*_*m*_ (mm)	*F*_*gy*_(kN)	*S*_*gy*_(mm)	*W*_*m*_(J)	*W*_*a*_(J)
25°C	1	139.16	16.41	22.64	2.55	17.7	1.076	38.48	100.68
	2	148.57	17.26	22.75	3.51	18.11	1.579	49.21	99.36
	3	145.62	1.98	2.64	0.314	2.48	0.262	0.45	145.17
	Average	144.45	11.88	16.01	2.12	12.76	0.97	29.38	115.07
−20°C	1	74.1	11.74	21.88	1.567	19.28	1.024	19.09	55.01
	2	75.91	11.96	20.95	1.882	18.19	1.369	17.64	58.27
	3	71.28	11.14	22.1	1.632	19.21	1.159	17.84	53.44
	Average	73.76	11.61	21.64	1.69	18.89	1.18	18.19	55.57
−60°C	1	71.53	8.87	22.23	1.66	19.16	1.086	19.96	51.57
	2	74.88	12.22	22.12	1.949	18.62	1.013	27.1	47.78
	3	77.21	9.04	22.82	2.106	18.91	1.18	27.81	49.4
	Average	74.54	10.04	22.39	1.91	18.90	1.09	24.96	49.58

Temperature, *T*; Sample number, S/N; Impact energy, *W*_*t*_; Total displacement, *S*_*t*_; Maximum force, *F*_*m*_; Displacement at maximum force, *S*_*m*_; Yield force, *F*_*gy*_; Displacement at yield force, *S*_*gy*_; Crack initiation energy, *W*_*m*_; Crack propagation energy, *W*_*a*_.

Table 2 and Table 3 respectively list the impact performance parameters of the joint samples at different temperatures. With the decrease of temperature, the impact energy decreases significantly, especially at −60 °C, which shows that the toughness of the material becomes worse with the decrease of temperature, and the impact toughness also decreases with the decrease of temperature. At −60 °C, the toughness of the joint material is the lowest, and the reduction of area also decreases at low temperature, indicating that the ductility and plasticity of the material are reduced at low temperature.

As shown in Fig 7 and Fig 8, the impact displacement curves and their corresponding impact energy curves of the transverse and longitudinal specimens under different temperature conditions. It shows that the specimens under normal temperature have high resistance at the initial stage of impact, the maximum impact force is about 29.2 kN, and the impact energy is relatively high, reaching about 130140 J. Under low temperature conditions, the maximum impact force and impact energy are reduced, the maximum impact force is about 25.8 kN, and the impact energy is about 100120 J, indicating that the toughness of the specimen is weakened. Under ultra-low temperature conditions, the impact force and impact energy are reduced to the lowest, the maximum impact force is about 23.2 kN, and the impact energy is only 7080 J. The material shows significant brittleness at low temperature, and the resistance to significantly reduced impact capacity.

### 2.3. Anti torsion performance test

**Fig 9:**
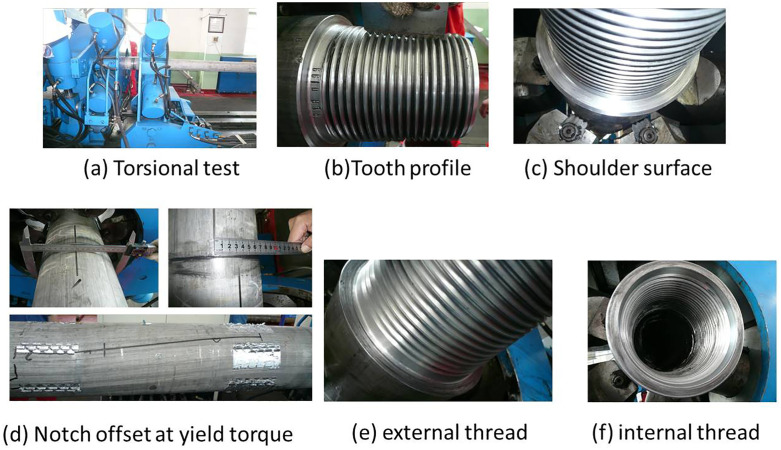
Torsional test process. (a) Torsional test,(b)Tooth profile,(c) Shoulder surface,(d) Notch offset at yield torque, (e) Wear mark of external thread, (f) Wear mark of internal thread.

During the torsional performance test, the Griffith TORQUEMASTER machine is used to simulate the torsional capacity of the joint under actual working conditions. The test sample is tightened to reach the predetermined tightening state, and the torque is gradually loaded from the make-up torque to the limit torque. After the test, the damaged parts were observed and recorded in detail to further analyze the failure mechanism and failure mode of torsional resistance. The test process is shown in Fig 9(a)-(f).

Under the yield torque, the thread profile and shoulder surface remain intact, and the position of the scoring line on the pipe surface is offset. Under the limit torque, obvious wear marks appear on the shoulder surface of the external thread and internal thread, indicating that the shoulder surface bears greater friction at the limit torque.

## 3. Mechanical analysis of thread joints

### 3.1. Analysis of torque on threads

**Fig 10:**
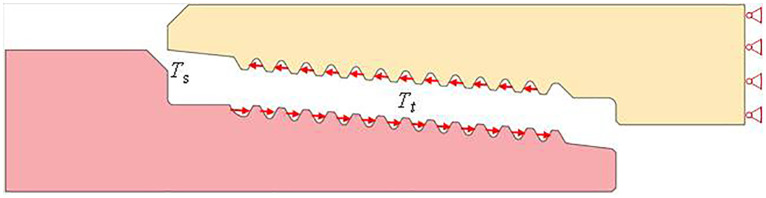
Force analysis of thread under preload condition.


The joint of drill pipe can tightly contact the threaded teeth and the shoulder surface by applying torque to withstand combined loads during operation. Appropriate tightening torque can ensure effective engagement and load transmission of threads, and also prevent loosening or failure during operation. As shown in Fig 10, apply pre tightening force to the drill pipe joint through the make-up torque *T*. Under the action of the make-up torque, the thread torque *T*_*t*_ is generated between the thread mating surfaces, and the friction torque *T*_*s*_ is generated between the shoulder contact surfaces. Therefore, the make-up torque *T* can be expressed as:

$$\begin{array}{c} T=T_{t}+T_{s}=\frac{SA}{12}\left(\frac{p}{2\pi }+\frac{R_{t}f}{cos\alpha }+R_{s}f\right) \end{array}$$ (1)

*A* is the actual effective contact area, mm^2^; *S* is the recommended makeup stress level, MPa; *P* is the pitch, mm; α is the half apex angle of the thread, °; *f* is the friction coefficient; *R*_*t*_ is the load radius on the thread tooth surface, mm; *R*_*s*_ is the load radius on the shoulder surface, mm.

In the screwing process of the threaded joint, the pre tightening force will be generated at the threaded joint, and the friction torque generated between the thread mating surface and the shoulder contact surface will hinder the loosening of the threaded joint. The relationship between the loosening torque and the pre tightening force can be expressed as:


$$\begin{array}{c} T_{L}=T_{tl}+T_{sl}=k\cdot d\cdot F \end{array}$$ (2)

$$\begin{array}{c} T_{tl}=\left(\frac{R_{t}}{cos\alpha }f+\frac{P}{2\pi }\right)F \end{array}$$ (3)

$$\begin{array}{c} T_{sl}=F\cdot f\cdot R_{s} \end{array}$$ (4)

Friction torque on the surface of thread teeth after the action of make-up torque *T*_*tl*_, N·mm; on the surface of thread after the action of make-up torque; friction torque *T*_*sl*_, N·mm; on the back shoulder surface after the action of make-up torque; *k* is the tightening coefficient, *d* is the pitch diameter of thread, mm; *F* is the pre tightening force, and the definition of tightening coefficient *k* is:


$$\begin{array}{c} k=\frac{\text{1}}{d}\left(\frac{R_{t}}{cos\alpha }f+\frac{P}{2\pi }+R_{s}f\right) \end{array}$$ (5)

After applying the make-up torque, the axial pre tightening force *F* will be generated at the engagement of the threaded joint. Among them, the friction torque *T*_*tl*_ of the thread tooth surface is converted into the friction torque required when the thread joint is loosened, while the friction torque *T*_*sl*_ of the shoulder contact surface is converted into the friction torque required to overcome the slip of the shoulder contact surface.

Thus, the expression of the total friction torque *T*_*L*_ required for thread joint loosening is:


$$\begin{array}{c} T_{L}=\left(\frac{R_{t}}{cos\alpha }f+\frac{P}{2\pi }+R_{s}f\right)F \end{array}$$ (6)

Because the threaded joint will produce complex stress distribution when bearing axial load, torque and internal pressure, including the superposition of axial tension, torsion, internal pressure and bending load, yield criterion of von Mises is selected to predict the failure behavior of isotropic materials.


$$\begin{array}{c} \sigma_{s}=\sqrt{\frac{\text{1}}{\text{2}}\left[{\left(\sigma_{\text{1}}-\sigma_{\text{2}}\right)}^{\text{2}}+{\left(\sigma_{\text{2}}-\sigma_{\text{3}}\right)}^{\text{2}}+{\left(\sigma_{\text{3}}-\sigma_{\text{1}}\right)}^{\text{2}}\right]} \end{array}$$ (7)

$$\begin{array}{c} \sigma_{s}\geq \sigma_{y} \end{array}$$ (8)

*σ*_*s*_ is the von Mises equivalent stress, MPa, *σ*_*y*_ is the yield strength of the material, MPa, *σ*_1_, *σ*_2_ and *σ*_3_ are the three principal stresses, MPa. When the equivalent stress exceeds the yield strength, the threaded joint will enter the plastic failure stage, which may cause damage to the threaded surface or joint failure.

After the thread joint is screwed, the shoulder surface of the male thread and female thread is cylindrical seal, and the contact pressure is *P*_1_ and *P*_2_ respectively. The contact pressure of the male and female thread on the contact surface can be calculated by Farr formula:


$$\begin{array}{c} P_{s}=\frac{12T}{\frac{h}{2\pi }+\frac{R_{t}f}{cos\theta }+R_{s}f} \end{array}$$ (9)

*P*_*s*_ is the contact pressure between the shoulder surfaces, MPa; *T* is the torque required for making up, N·mm; *h* is the pitch of the thread, mm; *R*_*t*_ is the average radius of the thread, mm; *f* is the friction factor between the shoulder surfaces of the male and female fasteners; *R*_*s*_ is the average radius of the shoulder, mm; *θ* is the half angle of the thread profile, °.

For NC38 and TM40 thread profiles, the geometry of the thread needs to be corrected. The contact surface of the thread is inclined. Therefore, the actual normal contact area needs to be calculated according to the thread angle, which can be corrected according to the height and angle of the thread profile:


$$\begin{array}{c} A_{\text{contact}}=\frac{P\cdot d_{\text{ef}}}{2sin\theta } \end{array}$$ (10)

*A*_*contact*_ is the actual normal contact area, mm^2^; *P* is the pitch, mm; *d*_*ef*_ is the effective contact surface diameter, mm; *θ* is the thread angle, °;

The thread tooth and shoulder face bear loads at the same time, so it is necessary to calculate the distribution ratio of axial load between the thread and shoulder face. Usually, the thread tooth bears most of the load, and the shoulder surface bears a smaller part:


$$\begin{array}{c} F_{thread}=a\cdot F_{axial} \end{array}$$ (11)

$$\begin{array}{c} P_{new}=\frac{F_{thread}\cdot 2sin\theta }{P\cdot d_{ef}} \end{array}$$ (12)

Take the load distribution coefficient *a* as 0.85, the axial force on the thread is *F*_*thread*_, N; the axial force on the end face of the pin is *F*_*axial*_, N; the corrected thread contact pressure is *P*_*new*_, MPa.

### 3.2. Bending analysis of thread joints

**Fig 11:**
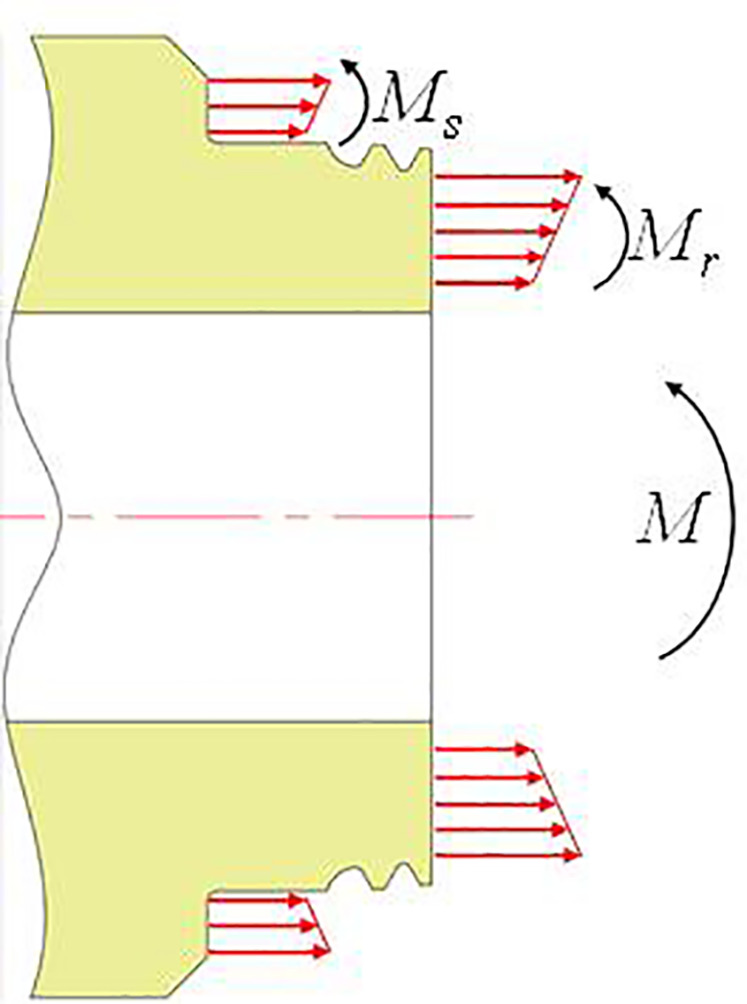
Bending diagram of pin.


As shown in Fig 11, the thread strength when the joint is subjected to bending moment. The proportion of the bending moment load borne by the shoulder surface, so according to this proportion:

$$\begin{array}{c} M=M_{s}+M_{r} \end{array}$$ (13)

$$\begin{array}{c} \frac{M_{s}}{M_{r}}=\frac{EJ_{s}}{EJ_{r}}=\frac{J_{s}}{J_{r}} \end{array}$$ (14)

The total bending moment of the pin is *M*, N·m, the bending moment of the shoulder surface is *M*_*s*_, N·m, the bending moment of root of the pin thread is *M*_*r*_, N·m, and *J*_*s*_ is the section moment of inertia of the shoulder surface; *J*_*r*_ is the moment of inertia of the root section of the male thread. So:


$$\begin{array}{c} M=M_{s}+M_{r}=M_{r}\left(1+\frac{J_{s}}{J_{r}}\right) \end{array}$$ (15)

And: $M=M_{s}\left(1+\frac{J_{r}}{J_{s}}\right)$

In order to ensure close contact between the shoulder surfaces during operation, the maximum bending stress *σ*_*max*_ of the shoulder caused by bending moment shall not exceed the pre pressure *σ*_1_, and the load distribution constant *a* can be used to express the proportion of bending moment:


$$\begin{array}{c} \sigma_{max}=a\sigma_{\text{1}} \end{array}$$ (16)

$$\begin{array}{c} \sigma_{max}=\frac{M_{s}}{W_{s}} \end{array}$$ (17)

$$\begin{array}{c} M_{s}=\frac{2J_{s}}{D_{\text{1}}}a\sigma_{\text{1}} \end{array}$$ (18)

$$\begin{array}{c} M_{r}=M_{s}\frac{2J_{r}}{D_{\text{1}}}a\sigma_{\text{1}} \end{array}$$ (19)

Ms-bending moment at the shoulder, N·m; Mr- The bending moment at the root of the pin, N·m.

The bending stress on the root of the pin and box can be calculated, the formula as follow:


$$\begin{array}{c} \sigma_{p}=\frac{M_{r}}{W_{r}}=\frac{R}{D_{\text{1}}}a\sigma_{\text{1}} \end{array}$$ (20)

$$\begin{array}{c} \sigma_{b}=\frac{M_{r}}{J_{s}}\cdot \frac{d_{\text{1}}}{\text{2}}=\frac{d_{\text{1}}}{D_{\text{1}}}\cdot \frac{J_{r}+J_{T}}{J_{s}}a\sigma_{\text{1}} \end{array}$$ (21)

$$\begin{array}{c} M=M_{s}+M_{r}+M_{\text{0}} \end{array}$$ (22)

*R* -section diameter, mm; *D*_1_ – outer diameter of the joint, mm; *d*_1_ – inner diameter, mm.

## 4. Establishment of finite element model

**Fig 12:**
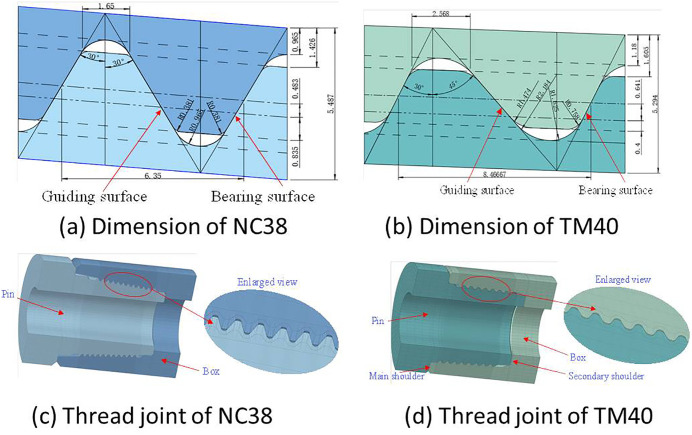
Finite Element Model. (a) Dimension of NC38 thread tooth,(b) Dimension of thread tooth of TM40, (c) Thread joint of NC38,(d) Thread joint of TM40.

In order to analyze the failure mechanism of drill pipe threaded joint, the finite element analysis method was used to analyze the stress distribution in the failure state. Considering the influence of thread rise angle, a three-dimensional finite element analysis model is established, as shown in Fig 12(a) and (b) are two types of thread joint, of which the taper of NC38 thread is 1:6, the taper of TM40 thread is 1:16, and Fig 12(c) and (d) are NC38 double shoulder thread joint and TM40 standard thread joint.

**Fig 13:**
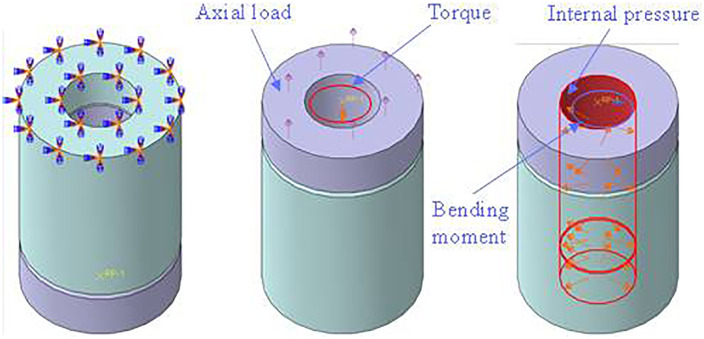
Boundary conditions and loads.

The box end face of the model adopts completely fixed boundary conditions is shown in Fig 13. The load is applied to the pin, and the end face of the large end of the pin bears axial load. At the same time, the end face is coupled to the center point, and torque and bending moment are applied at the coupling point.

## 5. Finite element analysis of NC38 threaded joint

### 5.1. Analysis of torsional results

**Fig 14:**
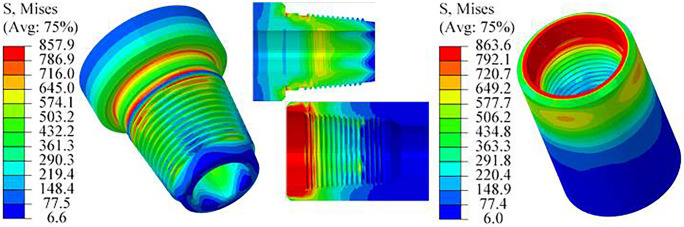
Stress contour of pin and box after fastening on a single shoulder structure.

**Fig 15:**
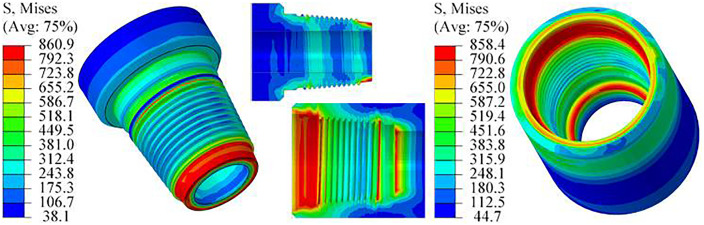
Stress contour of pin and box after fastening on a double shoulder structure.

Fig 14 and Fig 15 show the stress contour of NC38 thread joint respectively. Compared with the single shoulder joint, the high stress area of the big end of the double shoulder pin is reduced, and the high stress area of the main shoulder of the box is reduced.

**Fig 16:**
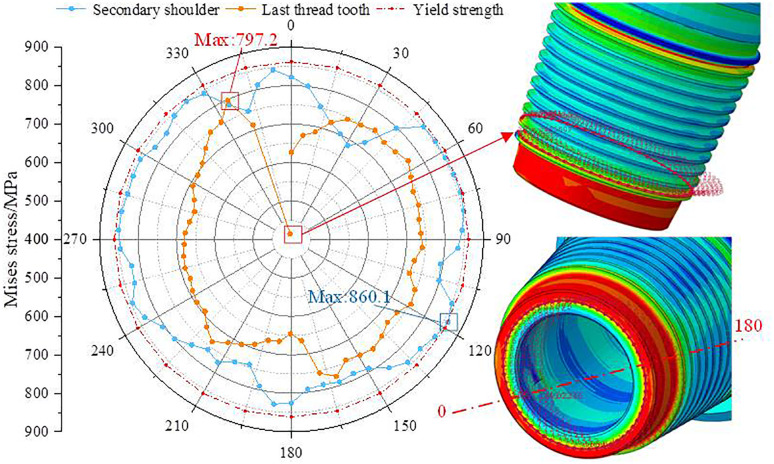
Stress curves of thread teeth of secondary shoulder.

Fig 16 shows the stress variation curves on the path of the joint of double shoulder structure. The overall stress value of the thread of the double shoulder is about 700MPa, the maximum stress value is 797.2MPa, and the maximum stress value of the secondary shoulder is 860.1MPa, which is close to its yield strength. Plastic deformation is easy to occur at this position.

**Fig 17:**
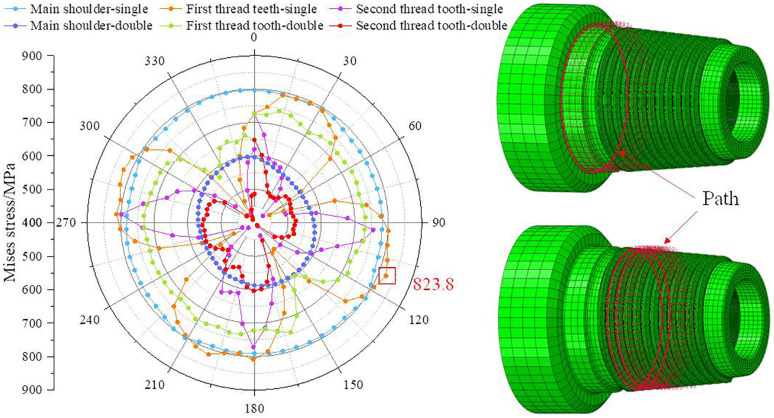
Stress variation curves on the path taken.

**Fig 18:**
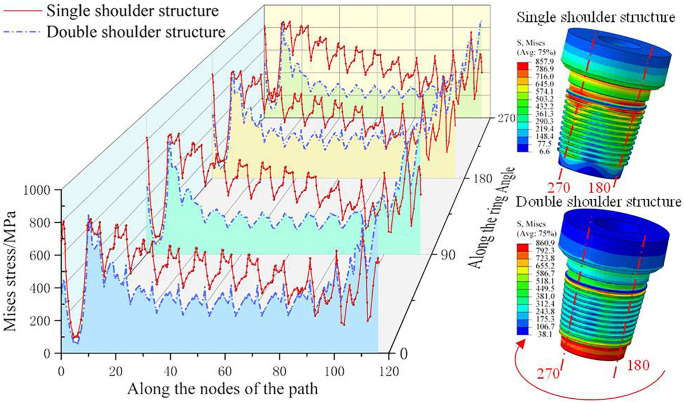
Stress variation curves on the axial path of thread teeth.

As shown in Fig 17, the stress variation curves along the paths of the joint of single shoulder and double shoulder are presented. The stress on the primary shoulder surface of the single shoulder structure approaches 800 MPa, while the double shoulder structure shows significantly lower stress on the primary shoulder. The high-stress regions on the thread teeth are regularly distributed, and the stress remains below the yield strength. The first thread of single shoulder presents the highest stress, reaching a maximum of 750 MPa, while the high-stress area on the second thread is notably smaller. Comparative analysis indicates that the double shoulder structure has higher strength and improved torsional resistance.As shown in Fig 18, stress values along axial paths at circumferential positions of 0°, 90°, 180°, and 270° are plotted. Along these paths, the double shoulder structure presents higher stress than the single shoulder structure only at the last thread. It shows the stress transfer across the secondary shoulder surface via the contact interface and material continuity, leading to stress concentration in the thread area. The maximum stress reaches 858.1 MPa, which could result in fatigue damage or fracture of the threads, potentially compromising the overall structural integrity and service life of the connection.

**Fig 19:**
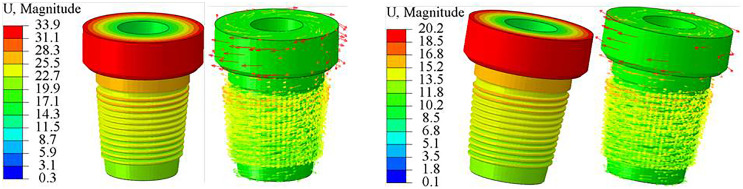
Displacement vector contour for the pin of the two structures.

The displacement of the pin is illustrated in Fig 19. Both structures present similar deformation patterns, but the single shoulder structure experiences greater overall displacement. The maximum displacement of the pipe body is 33.3 mm, with the thread section showing a deflection of approximately 25.5 mm, indicating significant bending under torque. In contrast, the double shoulder structure has a maximum pipe body displacement of 20.2 mm, with a thread deflection of around 15.2 mm. So the double shoulder demonstrates less bending compared to the joint of single shoulder.

**Fig 20:**
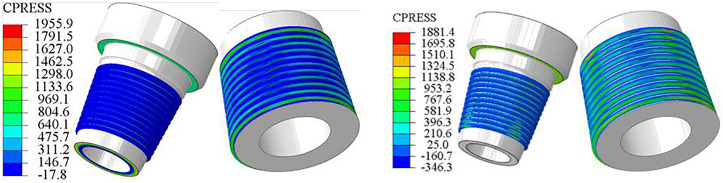
Contact pressure contour for the pin of the two structures.

The interference occurs between the bearing surfaces after deflection under torque in both structures in Fig 20. The significant interference fit generates a large preload, resulting in high contact pressure across most areas. Compared to the double shoulder structure, the single shoulder design presents more localized high-pressure regions. While the increased preload enhances the strength and load-bearing capacity of the joint, it also raises the risk of local wear and damage. The guiding surface provides lateral support and guiding functions, but the contact pressure distribution is uneven. There is a gap between the guiding surfaces of the pin and box, creating tensile forces and leading to negative contact pressure in some areas. This indicates that the guiding surface may bear lateral loads in certain regions, while the tensile forces arise due to assembly clearance in most areas.

**Fig 21:**
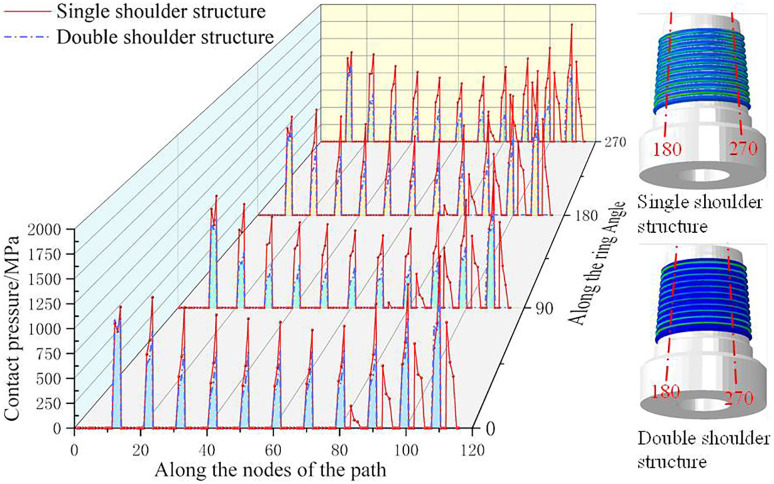
Variation curve of contact pressure in axial path of thread teeth.

The contact pressure on the thread teeth presents a periodic distribution along the circumferential direction, with localized high-pressure areas in Fig 21. The axial path contact pressure variation curve show that the double shoulder structure has lower contact pressure across all four paths compared to the single shoulder structure. Except for the first and last two threads, where the contact pressure reaches 1000 MPa, the maximum contact pressure in other regions does not exceed 800 MPa. In contrast, it shows the maximum contact pressure is 1250 MPa in the single shoulder structure. Such high contact pressure increases friction and can lead to material adhesion, which adversely affects the disassembly and service life of the threaded connection.

### 5.2. Combined load of axial tension—torque—internal pressure

**Fig 22:**
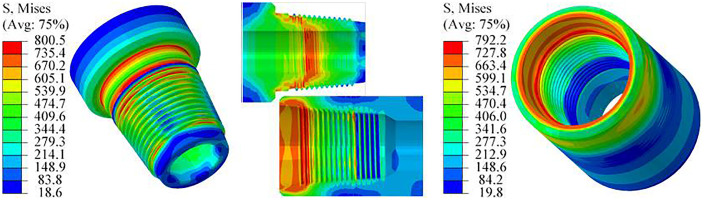
Stress contour of single shoulder structure under combined load.

**Fig 23:**
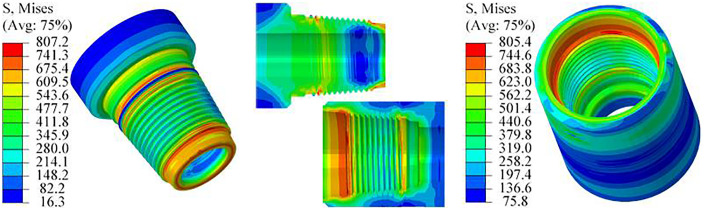
Stress contour of double shoulder structure under combined load.

Fig 22 and 23 are the stress contour for two types of drill pipe. The axial tensile load of 100 tonnes is applied to the pin end face after applying a 10 kN·m, along with an internal pressure of 100 MPa on the drill pipe. The pin with single shoulder presents a large high-stress area under combined loads, resulting in significant elastic deformation. At this point, the material may begin to undergo microscopic plastic deformation. Compared to the single shoulder structure, the double shoulder design shows a noticeable reduction in the high-stress region and features a more uniform stress distribution.

**Fig 24:**
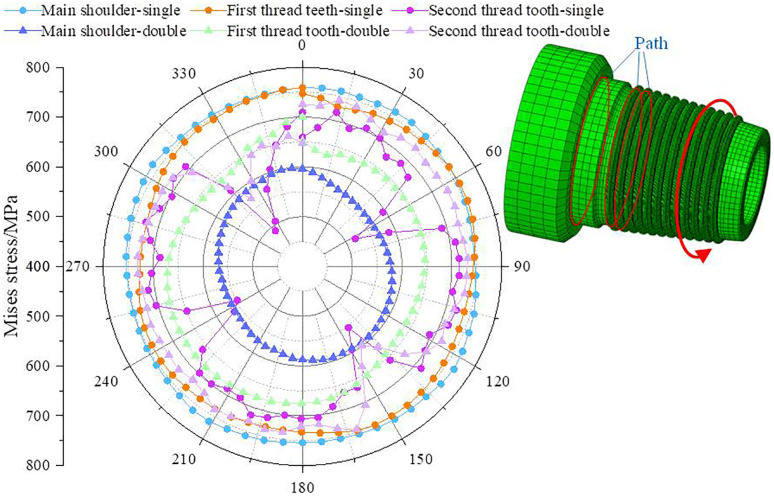
Stress variation curves of the two structures on the path of the pin.

As shown in Fig 24, the selected paths and stress variation curves for the pin with both structures are presented. The stress distribution on the primary shoulder and the first thread shows a uniform pattern for both designs, though the double shoulder structure presents lower stress levels. For the second thread, the stress distribution is similar in both structures with little numerical difference. The changes in the high-stress regions indicate that the double shoulder connection has a more uniform stress distribution at the large end of the pin.

**Fig 25:**
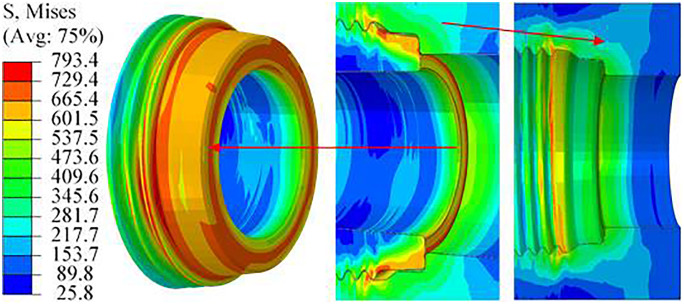
Stress contour at secondary shoulder of double shoulder structure.

The stress concentration occurs between at the secondary shoulder of the double shoulder in Fig 25, resulting in an overall stress level of 600 MPa on the outer wall of the pin’s. In contrast, the stress on the box is more uniform and remains in low-stress regions, staying within the elastic deformation range.

**Fig 26:**
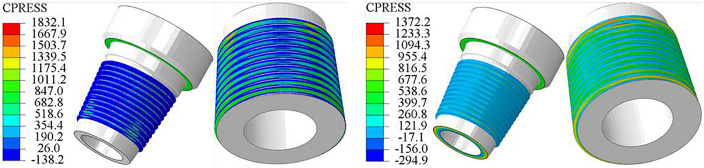
Contact pressure contour of single and double shoulder structures.

Fig 26 shows the contact pressure contour of the two structures under the combined load. The maximum contact pressure of the double shoulder is significantly lower than that of the single shoulder, and its contact pressure distribution is more uniform. At the same time, the contour shows that the contact pressure of the double shoulder structure presents an obvious periodic distribution along the thread path.

**Fig 27:**
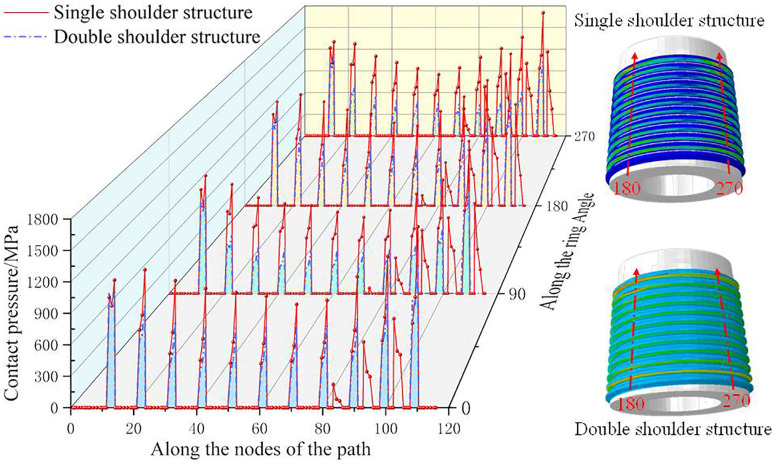
Contact pressure variation curves of the two structures on the pin.

At the 0°, 90°, 180°, and 270° positions along the pin thread, nodes in the axial direction are selected as paths, and contact pressure variation curves along these paths are plotted, as shown in Fig 27. For both structures, the contact pressure decreases from the large end to the small end, then gradually increases. The single shoulder structure shows a maximum contact pressure of 1832.1 MPa at the crest of the thread on the small end, which poses a risk of thread galling during disassembly. In contrast, the double shoulder structure has a lower maximum contact pressure of 1372.2 MPa, located on the second thread near the small end, with a more uniform pressure distribution.

**Fig 28:**
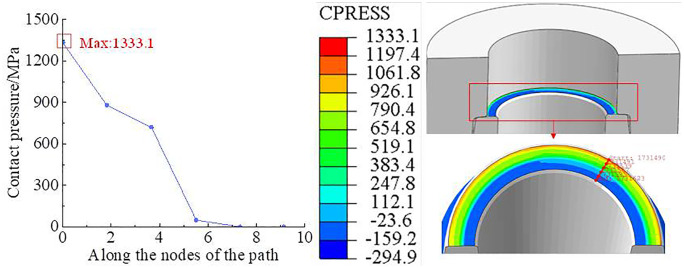
Contact pressure curves of secondary shoulder of double shoulder structure.

Fig 28 shows the contact pressure variation curve along the path at the secondary shoulder of the double shoulder structure. The contact pressure on the shoulder gradually decreases along the path, and reaches a maximum of 1333.1 MPa at the contact area between the two shoulder surfaces. As the path extends to the contact region between the box chamfer and the pin shoulder, the contact pressure drops to 719.8 MPa, then sharply decreases to 47.7 MPa. The elevated contact pressure near the shoulder edge may lead to material adhesion, affecting the sealing performance of the shoulder surface. Therefore, optimization is necessary to balance sealing performance and load-carrying capacity.

The overall stress level in the double shoulder connection is lower than the single shoulder structure, indicating higher strength under combined loading of torque. Additionally, the contact pressure distribution across the threads and shoulder surfaces is more uniform, ensuring tight thread engagement to effectively prevent leakage. Thus, the double shoulder structure has distinct advantages in complex and harsh conditions.

### 5.3. Combined load of axial tension—bending moment—torque—internal pressure

Under preload conditions, the double shoulder structure presents superior connection and sealing performance compared to the single shoulder structure after applying axial tension and internal pressure loads. To further evaluate the performance of drill pipe thread under bent wellbore conditions, it is necessary to analyze the effects of bending moments applied to the pin end face, in addition to the combined axial tension and internal pressure loads.

**Fig 29:**
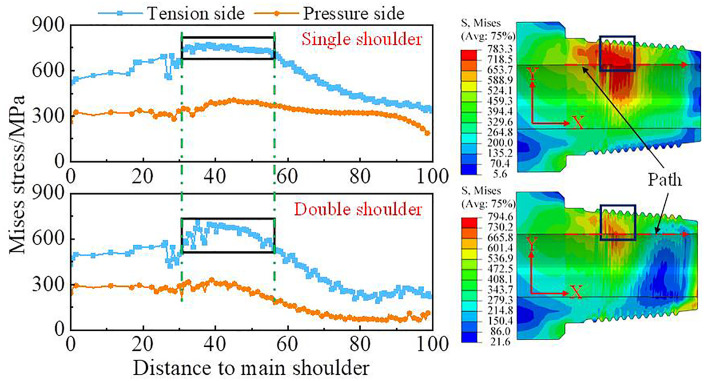
Stress curves of compression side and tension side.

As shown in Fig 29, the stress variation curves and stress contour for both structures on the compression and tension sides are presented. The stress levels of the two structures show the similar trends, and maintain low stress on the compression side. However, the double shoulder structure has significantly fewer high-stress regions on the tension side, and the overall stress level is lower than that of the single shoulder structure. The stress curves indicate that the internal surface stress increases gradually from the primary shoulder and reaches its peak in the thread region, where the double shoulder structure reaches a maximum of 693.8 MPa, while the single shoulder structure reaches 769.6 MPa.

**Fig 30:**
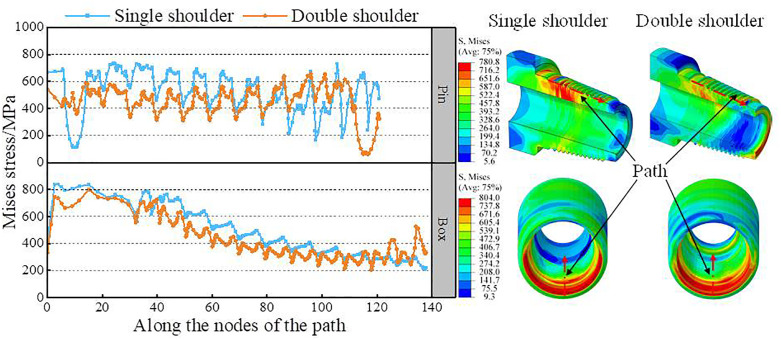
Stress curves change on thread teeth.

It shows the stress values on the tension side of the pin and box in Fig 30. The stress of the pin decreases from the root to the crest, and then increasing again from the crest to the root. There is a larger region with stress levels between 600 and 800 MPa in the first tooth of the single shoulder structure. The small-end region slightly exceeds 600 MPa in the double shoulder structure, resulting in lower overall stress levels and a more uniform distribution. On the compression side of the box, stress decreases from the primary shoulder surface and is periodically distributed along the thread teeth. The stress in the transition zone between the shoulder and the thread is relatively high, which could lead to plastic deformation if further increased.

**Fig 31:**
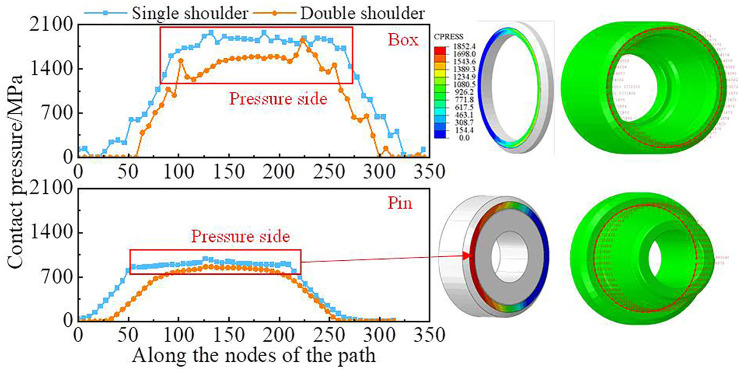
The contact pressure curves of the main shoulder.

Fig 31 is the contact pressure variation curves along the nodal path on the primary shoulder surface. The figure indicates a significant difference in contact pressure between the tension and compression sides of the shoulder surface, and the compression side of both the pin and box shoulders reaching approximately 1000 MPa. The contact pressure increases along the path from the tension side to the compression side and then gradually decreases. Both structures present similar trends in contact pressure variation, but the double shoulder structure shows lower overall contact pressure compared to the single shoulder structure, which helps prevent excessive pressure from causing material adhesion.

## 6. Finite element analysis of TM40 threaded joint

**Fig 32:**
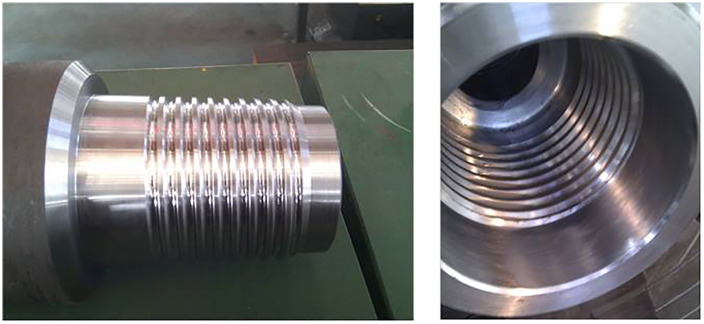
Pin and box of TM4.

In practical engineering applications, as the drilling depth increases and the requirements for complex downhole conditions arise, the NC38 thread may face a risk of failure under combined conditions. Especially in the stress concentration areas such as the root of the thread, fatigue cracks or plastic deformation are prone to occur [21]. To improve the performance of the double shoulder structure of NC38, the design of TM40 threaded joints was optimized. The extended structure features three non-engaging threads beyond the base of the pin, aimed at reducing the stiffness of the pin’s large end. Fig 32 shows the pin and box of the TM40. Numerical simulations were conducted to evaluate the connection and sealing performance of these two structures under combined tensile, bending and internal pressure loads.

**Fig 33:**
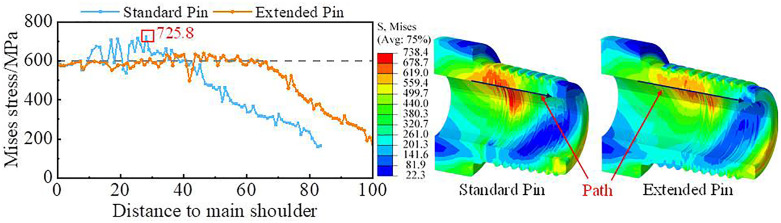
Stress variation curves of inner surface of tension side of pin.

Fig 33 is the stress curves of the two structures on the node path of the compression side. The stress in the front section of the standard structure is larger and the fluctuation is obvious, while the average stress in the corresponding position of the extended structure is lower and the fluctuation is small. With the increase of the distance from the shoulder surface, the stress of the two structures decreases gradually, and the change trend is the same.

**Fig 34:**
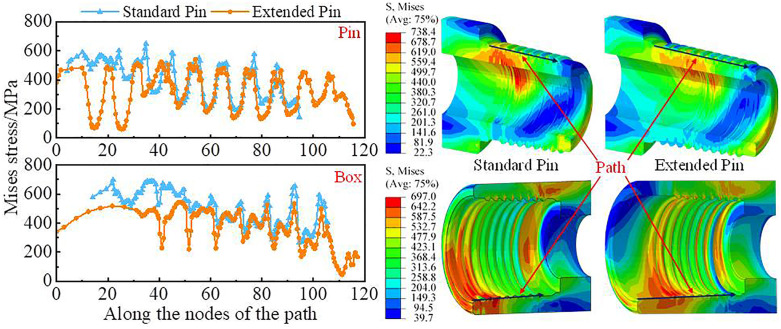
Curves of stress change on thread teeth.

As shown in Fig 34, the stress variation curves on the thread teeth correspond to the tensile side of the pin and the compressive side of the box. On the tensile side of the pin, both structures present a similar trend, with stress gradually increasing from the crest to the root of the thread. The stress levels on the extended thread are slightly lower than those on the standard thread. On the compressive side of the box, the extended thread presents a lower stress level, and the stress concentration localized in a small area on the main shoulder. The stress distribution along the threaded section is similar for both structure, but the extended thread demonstrates a more significant reduction in stress at the crest. Overall, the stress reduction at critical locations for the extended thread provides an advantage in enhancing the strength of the threaded connection under combined loading conditions.

**Fig 35:**
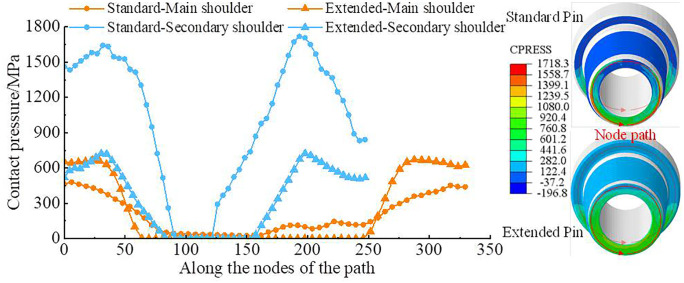
Curves of contact pressure variation on the shoulder of the pin.

As shown in Fig 35, the contact pressures on the primary and secondary shoulder surfaces for both structures were extracted, following a nodal path from the compression side around the circumference to the tension side and back to the compression side. For both structures, the contact pressure decreases initially, then reaching a minimum on the tension side and increases again on the compression side. The higher contact pressure on the compression side of the extended thread compared to the standard thread. On the primary shoulder surface, this is beneficial for sealing performance within safe limits. The contact pressure trend is similar, but the standard thread shows a larger variation on the secondary shoulder surface, with a maximum contact pressure of 1718.3 MPa. The pressure remains around 1500 MPa on the compression side but drops sharply along the path to the tension side. The standard thread shows significant differences in contact pressure between the primary and secondary shoulder surfaces.

**Fig 36:**
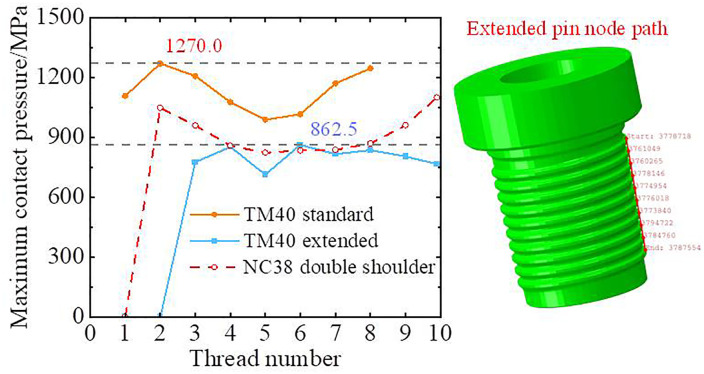
Variation curves of contact pressure on thread of pin.

The contact pressure data on the bearing surface of the compression side for both the standard and extended threads is as shown in Fig 36. The contact pressure reaches 875.6 MPa and remains around 900 MPa from the third to the tenth thread, and it ensures adequate sealing performance while reducing the risk of galling during assembly and disassembly. In contrast, the contact pressures are ranging from 900 to 1300 MPa of the standard thread. Excessive contact pressure at the second and eighth threads increases the likelihood of galling.

## 7. Conclusion

(1) It shows significant improvement in reducing stress concentration along the thread section after torsional preloading for the NC38 thread with a double shoulder structure, and the stress distribution on the thread was more uniform and maintained at a low level.
(2) The overall stress distribution of the double shoulder structure is more reasonable than that of the single shoulder structure when the axial tension and internal pressure combined loads are applied after pre tightening, and remains within the elastic deformation range. In addition, the contact pressure distribution of the threaded teeth of the double shoulder structure is more uniform, further reducing the risk of sticking.
(3) After increasing the bending moment load, the stress on the tensile side of pin thread of the NC38 joint with the single shoulder structure is larger, while it significantly reduces the high stress area on the tensile side and the stress on the thread on the tensile side for the double shoulder structure.
(4) It shows a more uniform stress distribution on the tensile side of the inner surface under combined loads for the TM40 joint with an extended thread. The stress distribution on the tensile side of both the pin and box threads is similar for the extended thread. The minimum stress value for the TM40 extended thread is 159.5 MPa, which is 26.4% lower than that of the NC38 double shoulder structure.
(5) The contact pressure distribution on the shoulder and thread surfaces of the TM40 joint is similar to that of the NC38 joint, but with lower overall contact pressure.

## Supporting information

S1 Data All the data in the curve graphs in the manuscript. (ZIP)
