# Supplementary material for: Performance analysis and structural optimization of drill pipe connections under complex operating conditions
Source: PLoS One. 2026 Aug 13;21(8):e0355727. doi: 10.1371/journal.pone.0355727 (PMC13472423; doi:10.1371/journal.pone.0355727)
Supplement: Peer review history

# Peer review: Decision Letter 0

**DOI:** 10.1371/journal.pone.0355727.r001

**Authors:** Van Thanh Tien Nguyen

18 May 2026

Dear Dr. Huang,

Thank you for submitting your manuscript to PLOS ONE. After careful consideration, we feel that it has merit but does not fully meet PLOS ONE’s publication criteria as it currently stands. Therefore, we invite you to submit a revised version of the manuscript that addresses the points raised during the review process.

Please submit your revised manuscript by Jul 02 2026 11:59PM. If you will need more time than this to complete your revisions, please reply to this message or contact the journal office at plosone@plos.org. When you're ready to submit your revision, log on to https://www.editorialmanager.com/pone/ and select the 'Submissions Needing Revision' folder to locate your manuscript file.

- A letter that responds to each point raised by the academic editor and reviewer(s). You should upload this letter as a separate file labeled 'Response to Reviewers'.
- A marked-up copy of your manuscript that highlights changes made to the original version. You should upload this as a separate file labeled 'Revised Manuscript with Track Changes'.
- An unmarked version of your revised paper without tracked changes. You should upload this as a separate file labeled 'Manuscript'.

If you would like to make changes to your financial disclosure, please include your updated statement in your cover letter. Guidelines for resubmitting your figure files are available below the reviewer comments at the end of this letter.

If applicable, we recommend that you deposit your laboratory protocols in protocols.io to enhance the reproducibility of your results. Protocols.io assigns your protocol its own identifier (DOI) so that it can be cited independently in the future. For instructions see: https://journals.plos.org/plosone/s/submission-guidelines#loc-laboratory-protocols. Additionally, PLOS ONE offers an option for publishing peer-reviewed Lab Protocol articles, which describe protocols hosted on protocols.io. Read more information on sharing protocols at https://plos.org/protocols?utm_medium=editorial-email&utm_source=authorletters&utm_campaign=protocols.

As the corresponding author, your ORCID iD is verified in the submission system and will appear in the published article. PLOS supports the use of ORCID, and we encourage all coauthors to register for an ORCID iD and use it as well. Please encourage your coauthors to verify their ORCID iD within the submission system before final acceptance, as unverified ORCID iDs will not appear in the published article. *Only*  the individual author can complete the verification step; PLOS staff *cannot*  verify ORCID iDs on behalf of authors.

We look forward to receiving your revised manuscript.

Kind regards,

Van Thanh Tien Nguyen, Ph.D.

Academic Editor

PLOS One

Journal requirements:

When submitting your revision, we need you to address these additional requirements.

1. Please ensure that your manuscript meets PLOS ONE's style requirements, including those for file naming. The PLOS ONE style templates can be found at

https://journals.plos.org/plosone/s/file?id=wjVg/PLOSOne_formatting_sample_main_body.pdf and

https://journals.plos.org/plosone/s/file?id=ba62/PLOSOne_formatting_sample_title_authors_affiliations.pdf

2. Thank you for stating the following financial disclosure:

“Guizhou Provincial Department of Science and Technology Guizhou Provincial Science and Technology Plan Project: 2024 Basic Research Program (Natural Sciences) Youth Guidance Project, Contract No.: Qiankehe Foundation-[2024] Youth 007.”

Please state what role the funders took in the study.  If the funders had no role, please state: "The funders had no role in study design, data collection and analysis, decision to publish, or preparation of the manuscript."

If this statement is not correct you must amend it as needed.

Please include this amended Role of Funder statement in your cover letter; we will change the online submission form on your behalf.

3. Thank you for stating the following in the Acknowledgments Section of your manuscript:

“The authors are grateful to financial support from Guizhou Provincial Department of Science and Technology Guizhou Provincial Science and Technology Plan Project: 2024 Basic Research Program (Natural Sciences) Youth Guidance Project, Contract No.: Qiankehe Foundation-[2024] Youth 007.”

We note that you have provided funding information that is currently declared in your Funding Statement. However, funding information should not appear in the Acknowledgments section or other areas of your manuscript. We will only publish funding information present in the Funding Statement section of the online submission form.

Please remove any funding-related text from the manuscript and let us know how you would like to update your Funding Statement. Currently, your Funding Statement reads as follows:

“Guizhou Provincial Department of Science and Technology Guizhou Provincial Science and Technology Plan Project: 2024 Basic Research Program (Natural Sciences) Youth Guidance Project, Contract No.: Qiankehe Foundation-[2024] Youth 007.”

Please include your amended statements within your cover letter; we will change the online submission form on your behalf.

4. PLOS requires an ORCID iD for the corresponding author in Editorial Manager on papers submitted after December 6th, 2016. Please ensure that you have an ORCID iD and that it is validated in Editorial Manager. To do this, go to ‘Update my Information’ (in the upper left-hand corner of the main menu), and click on the Fetch/Validate link next to the ORCID field. This will take you to the ORCID site and allow you to create a new iD or authenticate a pre-existing iD in Editorial Manager.

If the reviewer comments include a recommendation to cite specific previously published works, please review and evaluate these publications to determine whether they are relevant and should be cited. There is no requirement to cite these works unless the editor has indicated otherwise.

Please review your reference list to ensure that it is complete and correct. If you have cited papers that have been retracted, please include the rationale for doing so in the manuscript text, or remove these references and replace them with relevant current references. Any changes to the reference list should be mentioned in the rebuttal letter that accompanies your revised manuscript. If you need to cite a retracted article, indicate the article’s retracted status in the References list and also include a citation and full reference for the retraction notice.

[Note: HTML markup is below. Please do not edit.]

Reviewers' comments:

Reviewer's Responses to Questions

**Comments to the Author**

1. Is the manuscript technically sound, and do the data support the conclusions?

Reviewer #1: Yes

Reviewer #2: Yes

**********

2. Has the statistical analysis been performed appropriately and rigorously?

Reviewer #1: Yes

Reviewer #2: Yes

**********

3. Have the authors made all data underlying the findings in their manuscript fully available?

The PLOS Data policy

Reviewer #1: Yes

Reviewer #2: Yes

**********

4. Is the manuscript presented in an intelligible fashion and written in standard English?

Reviewer #1: Yes

Reviewer #2: Yes

**********

Reviewer #1: This paper takes the double shoulder drill pipe joint with NC38 and TM40 thread as the research object, establishes a three-dimensional finite element model, and adopts the method of combining experiments and numerical simulation to explore the failure mechanism under combined working conditions such as upper locking torque, axial load, internal pressure and bending moment. This work is quite outstanding, and the obtained conclusions have significant engineering practical value and have been verified by actual engineering applications. The article is well-argued.

1.However, there is no explanation given for choosing the double shoulder drill pipe joint with NC38 and TM40 thread profiles as the research object. If the author had carefully studied the literature, they would have found that the NC38 thread is an important component for wellbore connection. Its diameter is 3 1/2 inches, the thread profile is that of the drill pipe, the pitch is 3.175 millimeters, and the thread height reaches 1.524 millimeters. This thread design aims to ensure sealing performance, while also having good connection stability and shock resistance, making it the preferred choice in the oil drilling industry. And then, explaining the characteristics of the TM40 thread, and making these points clear, can demonstrate that this research has universal applicability value.

2.The abstract needs to be revised to better reflect the research content and innovation points of this paper.

3.Table 1 is not coherent from top to bottom and should be placed on one page.

Reviewer #2: This study addresses the performance analysis and structural optimization of drill pipe connections under complex operating conditions. By integrating theoretical analysis, numerical simulation, and experimental testing, it establishes three-dimensional finite element models of existing NC388 threaded drill pipe connections (single-shoulder and double-shoulder types) and conducts comparative analyses. The research further proposes a novel TM40 joint and evaluates the mechanical properties of its shoulder and threaded sections, demonstrating a certain degree innovation. The study closely aligned with practical engineering requirements, it hold both theoretical and practical importance for enhancing the reliability and safety of drill pipe connections. However, the following issues should be addressed before publication:

1. Are the material properties in the finite element model directly based on experimental averages, and does it account for the material’s hardening behavior? In this section, it should be clarified how the material parameters used in the model (e.g., elastic modulus, Poisson’s ratio, yield strength, etc.) were determined and their relationship to the experimental data should be explicitly stated.

2. The simulation should accurately replicate the actual constraints of the drill pipe within the wellbore, including accounting for the elastic support of surrounding rock formations or simulating wellbore wall resistance through multi-point support configurations rather than simple rigid fixation. Clearly specify the application method of loads (torque, bending moment)—whether directly applied to the end face or transmitted via coupling nodes—and indicate whether transmission efficiency during thread engagement is considered.

3. Where in the text are the conclusions mentioned in the abstract—such as “superior sealing performance” and “significant reduction in jamming risk” —specifically demonstrated? Please provide details. Additionally, these metrics lack quantitative evaluation or simulation. It is recommended to incorporate a leakage analysis by simulating fluid permeation under specific pressure differentials to quantify the sealing performance differences among various structures.

**********

what does this mean?). If published, this will include your full peer review and any attached files.

If you choose “no”, your identity will remain anonymous but your review may still be made public.

**Do you want your identity to be public for this peer review?**  For information about this choice, including consent withdrawal, please see our Privacy Policy

Reviewer #1: No

Reviewer #2: No

**********

[NOTE: If reviewer comments were submitted as an attachment file, they will be attached to this email and accessible via the submission site. Please log into your account, locate the manuscript record, and check for the action link "View Attachments". If this link does not appear, there are no attachment files.]

To ensure your figures meet our technical requirements, please review our figure guidelines: https://journals.plos.org/plosone/s/figures

You may also use PLOS’s free figure tool, NAAS, to help you prepare publication quality figures: https://journals.plos.org/plosone/s/figures#loc-tools-for-figure-preparation.

NAAS will assess whether your figures meet our technical requirements by comparing each figure against our figure specifications.

# Author comment: Author response to Decision Letter 1

**DOI:** 10.1371/journal.pone.0355727.r002

1 Jun 2026

Dear reviewers:

We are truly grateful to your and other reviewers’ critical comments and thoughtful suggestions on our manuscript. Based on these comments and suggestions, we have made careful modifications on the original manuscript. In addition, we have also made revisions and adjustments to the abstract and other parts of the manuscript.The red font indicates the modified content. So we would like to submit it to you for review again. We hope the new manuscript will meet your journal’s standard.

Response to Reviewer #1

Reviewer #1: This paper takes the double shoulder drill pipe joint with NC38 and TM40 thread as the research object, establishes a three-dimensional finite element model, and adopts the method of combining experiments and numerical simulation to explore the failure mechanism under combined working conditions such as upper locking torque, axial load, internal pressure and bending moment. This work is quite outstanding, and the obtained conclusions have significant engineering practical value and have been verified by actual engineering applications. The article is well-argued.

1.However, there is no explanation given for choosing the double shoulder drill pipe joint with NC38 and TM40 thread profiles as the research object. If the author had carefully studied the literature, they would have found that the NC38 thread is an important component for wellbore connection. Its diameter is 3 1/2 inches, the thread profile is that of the drill pipe, the pitch is 3.175 millimeters, and the thread height reaches 1.524 millimeters. This thread design aims to ensure sealing performance, while also having good connection stability and shock resistance, making it the preferred choice in the oil drilling industry. And then, explaining the characteristics of the TM40 thread, and making these points clear, can demonstrate that this research has universal applicability value.

Response: Thank you for this valuable comment. We have added detailed explanations to the section 6 of the revised manuscript.

2.The abstract needs to be revised to better reflect the research content and innovation points of this paper.

Response: Thank you for this valuable comment. We made revisions to the abstract.

3.Table 1 is not coherent from top to bottom and should be placed on one page.

Response: Thank you for this valuable comment. We have made the necessary revisions.

Response to Reviewer #2

Reviewer #2: This study addresses the performance analysis and structural optimization of drill pipe connections under complex operating conditions. By integrating theoretical analysis, numerical simulation, and experimental testing, it establishes three-dimensional finite element models of existing NC388 threaded drill pipe connections (single-shoulder and double-shoulder types) and conducts comparative analyses. The research further proposes a novel TM40 joint and evaluates the mechanical properties of its shoulder and threaded sections, demonstrating a certain degree innovation. The study closely aligned with practical engineering requirements, it hold both theoretical and practical importance for enhancing the reliability and safety of drill pipe connections. However, the following issues should be addressed before publication:

1. Are the material properties in the finite element model directly based on experimental averages, and does it account for the material’s hardening behavior? In this section, it should be clarified how the material parameters used in the model (e.g., elastic modulus, Poisson’s ratio, yield strength, etc.) were determined and their relationship to the experimental data should be explicitly stated.

Response: The material properties in the finite element model directly based on experimental averages. The stress-strain values obtained from the test were substituted into the material settings of the software to complete the calculation. Additionally, we considered hardening, but for the sake of simplifying the model, we adopted linear hardening (or ideal elastoplasticity and average yield strength). The joint material in this study is high-strength steel, which belongs to the stress distribution under small deformation. The material data is based on the real stress-strain curve (rather than the engineering stress-strain curve). Key parameters such as the hardening modulus/ strength coefficient/hardening index are calibrated through uniaxial tensile tests, and they cover the range of plastic strain from 0 to 0.2%.

2. The simulation should accurately replicate the actual constraints of the drill pipe within the wellbore, including accounting for the elastic support of surrounding rock formations or simulating wellbore wall resistance through multi-point support configurations rather than simple rigid fixation. Clearly specify the application method of loads (torque, bending moment)—whether directly applied to the end face or transmitted via coupling nodes—and indicate whether transmission efficiency during thread engagement is considered.

Response: In Fig.13 of this paper, the boundary conditions and load application are illustrated. Specifically, the axial load acts directly on the end face of the threaded connection. When torque or bending moment is applied to the end face, the load acts on the central node of the connection end face, and is transmitted to the entire end face area through rigid constraints or distributed coupling constraints. In the model, the transmission efficiency of the threaded connection is characterized by setting the thread contact friction coefficient (μ = 0.12).

3. Where in the text are the conclusions mentioned in the abstract—such as “superior sealing performance” and “significant reduction in jamming risk” —specifically demonstrated? Please provide details. Additionally, these metrics lack quantitative evaluation or simulation. It is recommended to incorporate a leakage analysis by simulating fluid permeation under specific pressure differentials to quantify the sealing performance differences among various structures.

Response: Thank you for your valuable suggestions. Through calculation, it is found that the contact pressure of the TM40 type threaded joint can seal the fluid pressure in the downhole as shown in Section 6 of this paper. Moreover, the average stress is also lower than that of the NC38 threaded structure. Therefore, the sealing performance of the TM40 joint is better. "Significant reduction in jamming risk" and "effectively reducing the risk of galling" are indeed not appropriate in the abstract and conclusion. They were not well considered, so these sentences have been deleted. Before the threaded part works downhole, it needs to undergo the thread engagement operation. During this period, lubricating grease needs to be applied to the threaded connection part, which can reduce the friction between the contact surfaces of the threaded teeth, reduce wear, extend service life, and enhance sealing performance. This paper mainly analyzes the variation law of contact stress of the threaded structure under the combined load from the perspective of mechanical properties. It is calculated that the contact pressure of the TM36 threaded structure is much greater than the fluid pressure difference in the downhole. Therefore, the sealing performance of the threaded joint can be guaranteed. In addition, during the operation of the threaded joint downhole, it is affected by various factors, and it is difficult to obtain the accurate critical leakage pressure and the curve of leakage rate with pressure difference. In the future, it can be compared and analyzed through indoor tests and on-site use to quantify the sealing performance differences between different threaded structures.

# Peer review: Decision Letter 1

**DOI:** 10.1371/journal.pone.0355727.r003

**Authors:** Van Thanh Tien Nguyen

26 Jul 2026

Performance analysis and structural optimization of drill pipe connections under complex operating conditions

PONE-D-25-66662R1

Dear Dr. Huang,

We’re pleased to inform you that your manuscript has been judged scientifically suitable for publication and will be formally accepted for publication once it meets all outstanding technical requirements.

Within one week, you’ll receive an e-mail detailing the required amendments. When these have been addressed, you’ll receive a formal acceptance letter and your manuscript will be scheduled for publication.

An invoice will be generated when your article is formally accepted. Please note, if your institution has a publishing partnership with PLOS and your article meets the relevant criteria, all or part of your publication costs will be covered. Please make sure your user information is up-to-date by logging into Editorial Manager at Editorial Manager® and clicking the ‘Update My Information' link at the top of the page. For questions related to billing, please contact billing support.

If your institution or institutions have a press office, please notify them about your upcoming paper to help maximize its impact. If they’ll be preparing press materials, please inform our press team as soon as possible -- no later than 48 hours after receiving the formal acceptance. Your manuscript will remain under strict press embargo until 2 pm Eastern Time on the date of publication. For more information, please contact onepress@plos.org.

Kind regards,

Van Thanh Tien Nguyen, Ph.D.

Academic Editor

PLOS One

Additional Editor Comments (optional):

Reviewers' comments:

Reviewer's Responses to Questions

**Comments to the Author**

Reviewer #2: All comments have been addressed

Reviewer #3: All comments have been addressed

**********

2. Is the manuscript technically sound, and do the data support the conclusions?

Reviewer #2: Yes

Reviewer #3: Yes

**********

3. Has the statistical analysis been performed appropriately and rigorously?

Reviewer #2: Yes

Reviewer #3: Yes

**********

4. Have the authors made all data underlying the findings in their manuscript fully available?

The PLOS Data policy

Reviewer #2: Yes

Reviewer #3: Yes

**********

5. Is the manuscript presented in an intelligible fashion and written in standard English?

Reviewer #2: Yes

Reviewer #3: Yes

**********

Reviewer #2: (No Response)

Reviewer #3: All concerns have been addressed. I suggest the revised manuscript could be accepted for publishing.

Thank you.

**********

what does this mean?). If published, this will include your full peer review and any attached files.

If you choose “no”, your identity will remain anonymous but your review may still be made public.

**Do you want your identity to be public for this peer review?**  For information about this choice, including consent withdrawal, please see our Privacy Policy

Reviewer #2: No

Reviewer #3: No

**********

# Editor report: Acceptance letter

**DOI:** 10.1371/journal.pone.0355727.r004

**Authors:** Van Thanh Tien Nguyen

PONE-D-25-66662R1

PLOS One

Dear Dr. Huang,

I'm pleased to inform you that your manuscript has been deemed suitable for publication in PLOS One. Congratulations! Your manuscript is now being handed over to our production team.

At this stage, our production department will prepare your paper for publication. This includes ensuring the following:

* All references, tables, and figures are properly cited

* All relevant supporting information is included in the manuscript submission,

* There are no issues that prevent the paper from being properly typeset

You will receive further instructions from the production team, including instructions on how to review your proof when it is ready. Please keep in mind that we are working through a large volume of accepted articles, so please give us a few days to review your paper and let you know the next and final steps.

Lastly, if your institution or institutions have a press office, please let them know about your upcoming paper now to help maximize its impact. If they'll be preparing press materials, please inform our press team within the next 48 hours. Your manuscript will remain under strict press embargo until 2 pm Eastern Time on the date of publication. For more information, please contact onepress@plos.org.

You will receive an invoice from PLOS for your publication fee after your manuscript has reached the completed accept phase. If you receive an email requesting payment before acceptance or for any other service, this may be a phishing scheme. Learn how to identify phishing emails and protect your accounts at https://explore.plos.org/phishing.

If we can help with anything else, please email us at customercare@plos.org.

Thank you for submitting your work to PLOS ONE and supporting open access.

Kind regards,

PLOS ONE Editorial Office Staff

on behalf of

Asst. Prof. Van Thanh Tien Nguyen

Academic Editor

PLOS One
